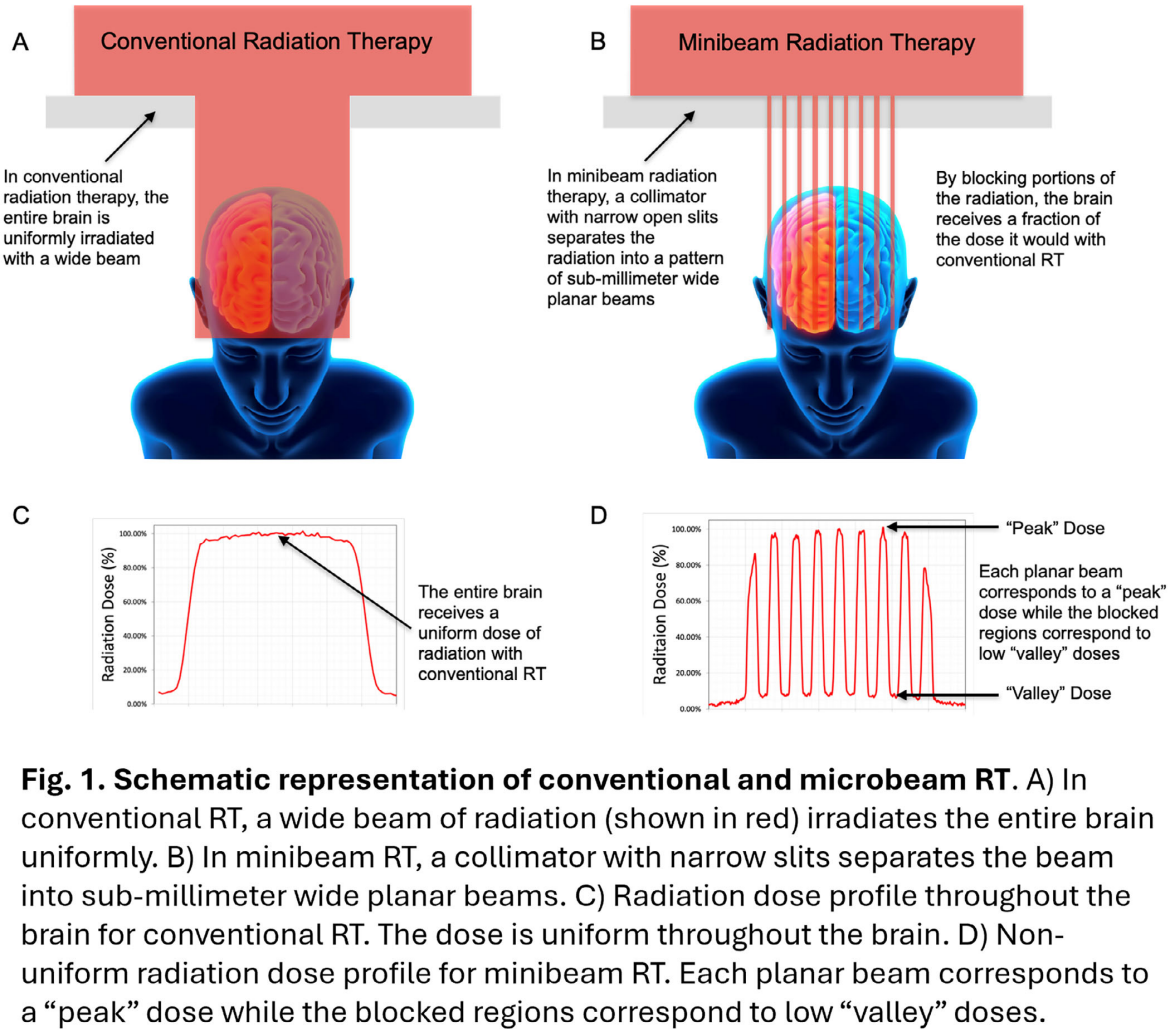# Preliminary Study of Low Dose Minibeam Radiation Therapy in a Murine Model of Alzheimer's Disease

**DOI:** 10.1002/alz70855_105186

**Published:** 2025-12-24

**Authors:** Jennifer Fazzari, Jarred Nesbitt, Eugenia Trushina, Michael Grams

**Affiliations:** ^1^ Mayo Clinic, Rochester, MN, USA

## Abstract

**Background:**

There is a critical need for novel treatment strategies addressing key features of Alzheimer's disease (AD) pathogenesis. Therapies targeting individual hallmarks yield little benefit. Low‐dose whole brain radiation is a non‐pharmacological approach that has shown promise in AD animal models, reducing amyloid plaques and neuroinflammation, and improving cognitive performance. These findings have initiated small clinical trials. However, even low doses (2 Gray per treatment) can cause adverse effects like vascular damage and reduced neurogenesis in the hippocampus.

Minibeam radiation therapy (MBRT) which delivers radiation in an alternating pattern of submillimeter‐wide high (“peak”) and low (“valley”) dose regions can potentially mitigate these negative effects. It has proven to reduce toxicity in a variety of normal tissues including the brain. Therefore, the objective of this study is to determine whether MBRT can safely and effectively address multiple AD mechanisms including amyloid deposition, neuroinflammation and brain energy metabolism while significantly reducing radiation exposure to the brain.

**Method:**

The study aims to develop an MBRT treatment paradigm to investigate its efficacy in targeting biochemical and cognitive hallmarks of AD. This includes assessing amyloid plaque burden, cognitive function, optimal treatment age, radiation dose, and duration of effect. In a preliminary study, 6‐month‐old 5XFAD mice were irradiated with MBRT (2 Gy peak and 0.3 Gy valley) or conventional irradiation (2 Gy) for a total of 5 daily treatments. Tissue was collected 4 weeks later for analysis of soluble and insoluble amyloid beta by ELISA and microscopic assessment of plaques stained with thioflavin S. Spontaneous alternation in the Y‐maze was assessed 4 weeks post‐irradiation.

**Result:**

Trends showing reductions in insoluble Aβ were observed in the brains of irradiated 5XFAD mice 4 weeks following MBRT. Although plaque burden is high in untreated 5XFAD mice, cognitive decline was not yet apparent at this age.

**Conclusion:**

Given these preliminary results, we will define optimal treatment parameters and assess efficacy when MBRT is administered after the onset of cognitive deficits.